# Simplified Light’s Criteria and Acute Phase Proteins Reflect Aetiology of Feline Body Cavity Effusions Better than the Traditional Classification Scheme

**DOI:** 10.3390/ani13121918

**Published:** 2023-06-08

**Authors:** Katarina Hazuchova, Susanne Held, Isabell Klemm, Natali Bauer

**Affiliations:** 1 Clinic for Small Animals (Internal Medicine, Clinical Pathology and Clinical Pathophysiology), Justus-Liebig-University of Giessen, 35392 Giessen, Germany; katarina.hazuchova@vetmed.uni-giessen.de (K.H.);; 2 Tierarztpraxis an der Erft, 50127 Bergheim, Germany

**Keywords:** acute phase proteins, biomarker, SAA, AGP, haptoglobin, effusion, Light’s criteria, albumin gradient, cat, feline

## Abstract

**Simple Summary:**

Analysis and classification of effusions is helpful in guiding diagnostic tests to identify an underlying disease. The traditional veterinary classification of effusions does not adequately reflect the disease mechanism causing the effusion in cats and might not sufficiently inform diagnostic work-up. The aim of this study was to assess whether other parameters might aid classification. Sixty-five cats with body cavity effusions (within the abdomen, chest and pericardial sac) were included. Effusions were classified as transudates (e.g., heart failure [*n* = 18]) or exudates (e.g., inflammation, cancer [*n* = 47]) based on the disease mechanism causing the effusion, and using the traditional scheme. Several parameters (activity of enzyme lactate dehydrogenase in the effusion; effusion/serum ratio of the enzymatic activity of lactate dehydrogenase; effusion/serum total protein ratio; serum–effusion albumin gradient; acute phase proteins in serum and effusion) were analysed in their ability to differentiate exudates from transudates. All tested parameters performed better in classifying effusions in comparison to the traditional scheme. Acute phase proteins were helpful in classifying effusions based on their disease mechanism but could not separate effusion types when using the traditional scheme. This latter finding further supports the classification of effusions based on their disease mechanism. Better classification of effusions might improve disease diagnosis in the future.

**Abstract:**

The traditional veterinary classification (TVC) of effusions based on cell count and total protein (TP) does not adequately reflect the aetiology. Light’s criteria (LC) (activity of lactate dehydrogenase [LDH] in the effusion [LDHef], effusion/serum LDH ratio [LDHr], effusion/serum TP ratio [TPr]), serum–effusion albumin gradient (ALBg), acute phase proteins (APPs) [serum amyloid A (SAA), α_1_-acid glycoprotein (AGP), haptoglobin] might aid classification. The aim was to evaluate the utility of these parameters except LDHr in differentiating exudates from transudates. Sixty-five cats with effusions (33 peritoneal, 31 pleural, 1 pericardial), with 18 transudates and 47 exudates based on aetiological classification (AC), were included. The sensitivity, specificity and accuracy of several parameters to identify exudates (based on AC) was assessed. APPs were compared between exudates and transudates based on AC and TVC, with receiver operating characteristics analysis identifying the best APP to recognise exudates. Simplified LC (LDHef, TPr) had an accuracy of 79% and TVC of 48%. ALBg had the highest sensitivity (98%) and LDHef the highest specificity (83%) in identifying exudates in cats. All APPs but effusion SAA could differentiate exudates from transudates based on AC (effusion AGP had the largest area under the curve 0.79) but not TVC. All parameters were better than TVC in identifying exudates. The conformity of APPs with AC but not TVC favours the use of AC to classify effusions.

## 1. Introduction

Cats with body cavity effusions are commonly presented in clinical veterinary practice. Characterisation of the effusion based on total nucleated cell count (TNCC) and total protein (TP) has been traditionally used in veterinary medicine to differentiate between transudates, modified transudates and exudates [1]. This classification is then used to narrow down potential differential diagnoses and guide the choice of appropriate diagnostic tests. For example, in a cat with pleural transudate and serum albumin/protein concentration within the reference range (RR), cardiac disease is considered the most likely differential, making echocardiography one of the initial tests in the diagnostic work-up [2,3]. However, effusions classified as modified transudates impose a certain diagnostic challenge, as they can originate from a variety of conditions, including cardiac disease, neoplasia or feline infectious peritonitis (FIP) [2]. While effusions caused by cardiac disease (i.e., congestive heart failure [CHF]) are unequivocally transudates based on their pathogenesis (i.e., increased hydrostatic pressure), increased capillary or mesothelial permeability plays a role in the pathogenesis of neoplastic effusions or those caused by FIP, which are therefore exudative in their nature [2,4,5]. This discrepancy between the classification method and the pathogenesis of the effusion calls the usefulness of this traditional veterinary scheme into question.

Indeed, the concept of the traditional veterinary classification of body cavity effusions in cats has recently been challenged [6,7]. Instead, the use of Light’s criteria to differentiate between exudates and transudates, adopted from human medicine [8], has been proposed, and cut-offs for feline species established [6]. These criteria include the activity of lactate dehydrogenase (LDH) in the effusion (LDHef), effusion/serum LDH ratio (LDHr), and effusion/serum TP ratio (TPr), although some authors have criticised the concurrent use of LDHef and LDHr as these two parameters are not independent from each other [9]. Furthermore, serum–effusion albumin gradient (ALBg) has been used in addition to Light’s criteria, especially in cases with discordant LDHef, LDHr and TPr results (e.g., LDHef and LDHr indicating transudate and TPr being suggestive of exudate) [10]. Similarly to human medicine, Light’s criteria performed better at differentiating exudates from transudates in the initial cohort of cats than in the second, validation cohort [6,7]. Both studies, however, were relatively small (20 and 19 cats) and only included cats with pleural effusions. Although Light’s criteria had originally been developed to classify thoracic effusions, these have been used in humans with ascites as well [11,12].

Acute phase proteins (APPs) are non-specific markers of inflammation, the concentration of which increases in response to various stimuli such as infections, non-infectious inflammatory conditions, trauma or neoplasia [13]. In cats, most studies evaluated the two major APPs, serum amyloid A (SAA) and α_1_-acid glycoprotein (AGP), and moderate APP haptoglobin (HP) [14]. Their concentration has been shown to increase in cats with various inflammatory and neoplastic conditions [15,16,17,18,19]. Due to their nature as non-specific inflammatory markers, the measurement of APPs might be useful in differentiating exudative from transudative effusions; however, this has not yet been assessed in cats.

This study had two objectives: firstly, to assess the diagnostic accuracy of several classification schemes to differentiate between exudates and transudates in a larger cohort of cats with body cavity effusions; and secondly, to assess the value of measurement of serum and effusion APPs in differentiating between exudates and transudates using aetiological classification and the traditional veterinary scheme.

## 2. Materials and Methods

### 2.1. Study Cats and Diagnostic Tests

This study used serum and effusion samples from a previous investigation [20,21,22]. Cats included in this study were presented to the Clinic for Small Animals of Justus-Liebig-University Giessen, Germany, over a period of two years. Samples were collected as part of diagnostic investigations from cats that presented a variety of clinical signs (dyspnoea, enlarged abdomen, fever, anorexia, lethargy, etc.), in which a pleural, peritoneal or pericardial effusion, or a combination thereof, was identified. In cats presented with more than one effusion, only one effusion was analysed in the present study. For inclusion in the study, a minimum of five millilitres of effusion had to be obtained. Cats treated by their referring veterinarian prior to presentation were not excluded.

All cats underwent routine haematology and biochemistry as well as FeLV/FIV testing (SNAP FIV/FeLV Combo Test, IDEXX Laboratories, Westbrook, ME, USA), with samples taken within one hour of effusion collection (see Section 2.2).

Analysis of the effusion is described below (Section 2.3). Other diagnostic tests, such as thoracic or abdominal radiographs or computed tomography, echocardiography or abdominal ultrasound were performed as recommended by the attending clinician. Diagnosis of CHF was made based on the presence of corresponding clinical signs (tachypnoea/dyspnoea), thoracic radiographs (where available), and echocardiography performed by a board-certified cardiologist or a resident under supervision [23]. Septic effusions were diagnosed based on cytology and culture results. Effusions secondary to neoplasia were diagnosed via cytology or histological examination. As described previously [20,21,22], several diagnostic tests were performed with reference to FIP. The diagnosis of FIP was made via immunohistochemistry where available (6/14 FIP cases included in this study), and by means of a sophisticated statistical method using machine learning in the remaining cases (8/14 FIP cases included in this study) [21].

The final diagnosis was confirmed via post-mortem examination in 20 cats (6/20 had FIP). All diagnostic test results pertinent to this study and the final diagnoses in all 65 cats can be found in Supplementary Material S1.

### 2.2. Haematology and Biochemistry

Complete blood count was performed using an automated analyser (ADVIA 2120, Siemens Healthcare Diagnostics, Eschborn, Germany). Blood smears were examined by experienced laboratory technicians if requested by the clinical pathologists validating the results. Biochemistry parameters were measured in heparin plasma on an automated analyser (ABX Pentra 400, Axonlab, Reichenbach/Stuttgart, Germany), using reagents provided by the manufacturer. Parameters specifically evaluated in this study included TP and albumin, which were both used to calculate ratios needed for the classification of the effusions (see Section 2.3). Total protein was measured using biuret reaction, and albumin concentration was determined via the colorimetric method using bromocresol green. An internal quality control was performed daily for both TP and albumin with commercially available material supplied by the manufacturer in two levels (normal and abnormal).

### 2.3. Analysis of the Effusion

The effusions were placed into K_3_-EDTA tubes and plain tubes and analysed within two hours of collection. Where effusions were collected out of hours, smears for cytological evaluation were prepared within two hours, and the remaining samples were placed in the refrigerator for analysis on the next working day. Analysis of the effusions included the measurement of TNCC (ADVIA 2120, Siemens), TP and albumin concentration, and LDH activity, as well as a cytological examination performed by a board-certified specialist in clinical pathology or under their supervision. Total protein, albumin and LDH activity were all measured on an automated analyser (ABX Pentra 400, Axonlab) using reagents provided by the analyser manufacturer. Total protein and albumin concentration were determined using the same methods as described above (Section 2.2). Activity of LDH was determined using an optimised ultraviolet (UV) test, which measures LDH activity with the reaction: $L-Lactate+{NAD}^{+}\overset{LDH}{⟷}Pyruvate+NADH+H^{+}$. The reaction was read at the wavelength of 340 nm (primary wavelength) and 420 nm (secondary wavelength). An internal quality control was performed daily for TP, albumin and LDH as described above (Section 2.2).

### 2.4. Measurement of APPs

Two major (SAA, AGP) and one moderate (HP) APPs were measured in effusion (SAAef, AGPef, HPef) and serum (SAAs, AGPs, HPs) samples in this study as described previously [22]. Briefly, SAA and HP were measured with an automated analyser ABX Pentra 400 (Axonlab) using a latex-agglutination method (LZ Test ‘Eiken’ SAA, Eiken Chemical, Tokyo, Japan) and a colorimetric assay (Phase Range Haptoglobin Kit [Second Generation], Tridelta Development, Maynooth, Ireland), respectively. Internal quality control material in two levels (normal and abnormal) provided by assay manufacturers was run for SAA and HP each prior to the analysis of patient samples. Alpha-1-acid glycoprotein was measured using single radial immunodiffusion (Phase Feline α 1 Acid Glycoprotein SRID Assay Kit, Tridelta Development, Maynooth, Ireland). All tests have been previously used for the measurement of APPs [16,24,25,26,27] and were performed according to the manufacturer’s instructions. The samples were stored at −80 °C for up to two years prior to measurement in batches. Good long-term stability of APPs has been shown in previous research [28].

### 2.5. Classification of Effusions

Effusions were classified based on their pathogenesis as transudates, resulting from reduced colloid osmotic pressure or increased hydrostatic pressure, and exudates, caused by increased capillary/mesothelial permeability [29]. Based on aetiological classification, considered the “gold standard” in this study, transudates were effusions secondary to CHF, other diseases leading to increased hydrostatic pressure (acute kidney injury [AKI]) or severe hypoalbuminaemia/panhypoproteinaemia. Exudates were effusions caused by FIP, septic or non-septic inflammation, neoplasia, bleeding and idiopathic chylothorax. Chylous effusions, diagnosed based on effusion/serum triglyceride ratio > 1 [30], were classified according to aetiology as transudates if they occurred secondary to CHF, or exudates if they were caused by neoplasia or idiopathic chylothorax [31].

**Figure 1:**
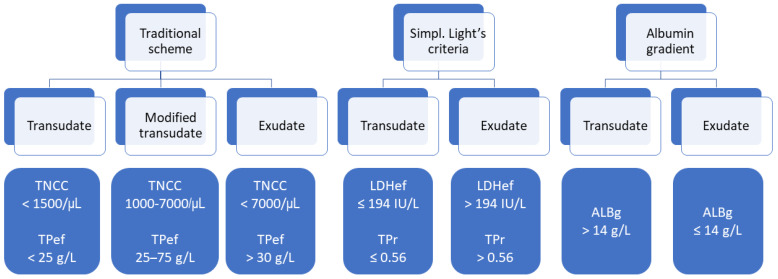
Classification of the effusions based on the traditional veterinary scheme, simplified (simpl.) Light’s criteria, and albumin gradient. TNCC—total nucleated cell count; TPef—total protein (effusion); LDHef—lactate dehydrogenase (LDH) activity (effusion); TPr—effusion/serum total protein ratio; ALBg—serum–effusion albumin gradient.

Effusions were further classified based on the traditional veterinary scheme as transudates, modified transudates, and exudates, depending on the TNCC and TP of the effusion (Figure 1) [1]. The classification of effusions as transudates or exudates was also undertaken using simplified Light’s criteria (LDHef, TPr), LDHef alone, TPr alone, and ALBg (Figure 1). The cut-offs for TPr > 0.56 and ALBg ≤ 14 g/L indicating exudates were adopted from previous studies [6,7]. For LDHef, the laboratory’s own LDHef cut-off was used because a different LDHef assay was used in this study (see above Section 2.2) compared to the previous investigations [6,7]. Because RR for serum LDH has not been established in the authors’ laboratory, it was not possible to calculate the LDHef cut-off using the two-thirds of the normal upper limit for serum LDH as used by others [32]. The laboratory’s own LDHef cut-off (LDH > 194 U/L indicating exudate) was calculated using data from 64 cats presented to the Clinic for Small Animals of the Justus-Liebig-University Giessen between April 2021 and December 2022 that were not included in the present study (see Supplementary Material S2 for information on LDHef cut-off calculation). In that dataset, LDHef > 194 U/L had a sensitivity and specificity of 82% (confidence interval [CI] 66.5–92.5%) and 100% (CI 86–100%), respectively, at identifying exudates. As described below (Section 2.6) for other parameters evaluated in the present study, the cut-off for LDHef was selected to maximise both sensitivity and specificity to differentiate between exudates and transudates. The third Light’s criterion, LDHr, was not used in the present investigation because LDH activity in blood was not measured. In agreement with previous feline studies [6,7] and human medicine literature [8], an effusion was classified as exudate when at least one of Light’s criteria (LDHef or TPr in the present study) indicated the presence of an exudate. The so-called discordant exudates (LDHef alone or TPr alone, but not both, indicating exudate) were treated as exudates in this study.

### 2.6. Statistical Analysis

Data were tested for normality using visual inspection of histograms and Shapiro–Wilk Tests. Due to their non-normal distribution, numeric data are presented as median (range). Categorical variables are presented as proportions (%). For statistical analysis, transudates and modified transudates based on the traditional veterinary scheme were grouped together as “transudates”, in agreement with previous studies [6,7], and also given their pathogenesis (modified transudates result from increased hydrostatic pressure) [33].

Sensitivity, specificity and accuracy of the traditional veterinary scheme, simplified Light’s criteria, LDHef alone, TPr alone, and ALBg in identifying exudates when compared to the “gold standard” aetiological classification of effusions were calculated as follows:

- $Sensitivity=\frac{Tp}{Tp+Fn}$
- $Specificity=\frac{Tn}{Tn+Fp}$
- $Accuracy=\frac{Tp+Tn}{Tp+Tn+Fp+Fn}$

In these equations, *Tp* is the number of true positive diagnoses, *Tn* is the number of true negative diagnoses, *Fp* is the number of false positive diagnoses, and *Fn* is the number of false negative diagnoses.

Concentrations of the three APPs in effusion and serum samples were compared between transudates and exudates classified based on aetiological classification and the traditional veterinary scheme using the Mann–Whitney U Test. To assess whether APPs (in serum or effusion) correspond better with aetiological or traditional classification, and determine which APPs are most useful in differentiating between exudates and transudates, receiver operating characteristics (ROC) analyses were performed by plotting sensitivity against (100-specificity). For all three APPs in effusion and serum samples with respect to both aetiological and the traditional veterinary classification, the areas under the curve (AUCs) were calculated to evaluate how well APPs can distinguish between exudates and transudates. Optimal cut-offs (to maximise both sensitivity and specificity) to differentiate between exudates and transudates were established for each APP for both classification systems. Within each classification system, the AUCs were compared using the method of Hanley and McNeil [34,35] to identify the parameter that can best differentiate exudates from transudates.

Because the number of samples that were used in this study was limited by the availability of suitable cases within the timeframe of the study and was not based on a power calculation, the power was calculated retrospectively for all APPs measured in serum and effusion samples. Power was calculated as 1-β error probability, using sample effect size (Cohen’s d) calculated from the U-values derived from the Mann–Whitney U tests and sample size. Sample effect sizes of d = 0.2, 0.5, and 0.8 were considered small, medium, and large, respectively [36]. Power ≥ 80% was considered sufficient.

Statistical analysis was performed using SPSS ver. 28 (IBM Statistics, Chicago, IL, USA), MedCalc ver. 20.215 (MedCalc Software Ltd., Ostend, Belgium) (ROC analysis) and G*Power 3.1.9.2 [37,38] (post hoc power analysis for two-tailed *t*-tests). Graphs were plotted using GraphPad Prism ver. 9.5.1 (GraphPad Software, San Diego, CA, USA) and MedCalc ver. 20.215 (ROC analysis).

## 3. Results

### 3.1. Study Cats

For this study, 65 cats with body cavity effusions described in a previous publication including 88 cats were used [22]. Twenty-three cats of the previous dataset were excluded because APPs were only measured in serum and not in effusion (*n* = 21) or due to the inability to make a clear aetiological diagnosis of the origin of the effusion (*n* = 2). A total of 5 out of the 65 cats had both pleural and peritoneal effusion collected as a part of the previous investigation; however, only ascites was included in the present study due to the lack of LDH and albumin measurement in all five pleural effusions.

The median age of the 62 cats included was 8.5 years (range 0.4–17.9); in 3 cats, the age was unknown. In total, 44 out of 65 cats were male (40 neutered, 4 intact), 20/65 were female (17 neutered, 3 intact), and the sex was not recorded in 1 cat. A total of 46 cats were Domestic Shorthair, and there were 19 pedigree cats (Maine Coon [*n* = 6], Persian [*n* = 4], British Shorthair [*n* = 3], Chartreaux [*n* = 2], and 1 each of Abyssinian, Birman, Ragdoll and Siamese).

### 3.2. Classification of Effusions

Of the 65 cats, 33 had ascites, 31 had pleural effusion, and 1 had pericardial effusion. Based on aetiology, there were 18 transudates secondary to increased hydrostatic pressure, but none due to reduced oncotic pressure; 47/65 effusions were exudates. The distribution of cats based on aetiology and localisation of the effusion is provided in Table 1. A total of 4 of the 65 cats had a chylous pleural effusion, of which 2 were classified as transudate secondary to CHF, and 2 were classified as exudate secondary to idiopathic chylothorax.

**Figure 2:**
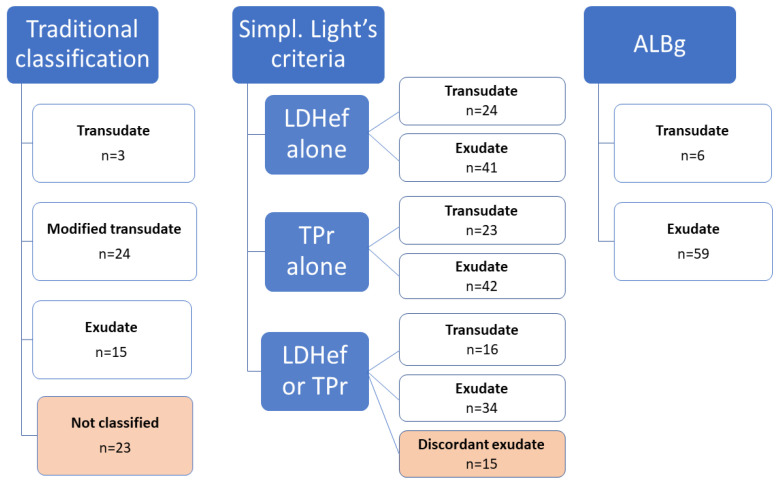
Classification of the 65 effusions (33 peritoneal, 31 pleural, 1 pericardial) based on the traditional veterinary scheme, lactate dehydrogenase activity in the effusion (LDHef), effusion/serum total protein ratio (TPr), simplified (simpl.) Light’s criteria (LDHef, TPr) and serum–effusion albumin gradient (ALBg).

The classification of the 65 effusions based on the traditional veterinary scheme, LDHef alone, TPr alone, simplified Light’s criteria and ALBg is shown in Figure 2.

The sensitivity, specificity, and accuracy of the traditional veterinary scheme, LDHef alone, TPr alone, simplified Light’s criteria, and ALBg to identify an exudate when compared to the “gold standard” aetiological classification is summarised in Table 2. The number (proportion) of the misclassified transudates and exudates is also provided.

Both pleural and peritoneal effusions were misclassified by all criteria, apart from exudates misclassified by ALBg (this was only one effusion, which was pleural). Given the low number of cases, statistical comparisons were not made to assess whether one effusion type (pleural or peritoneal) was misclassified more frequently. The traditional veterinary scheme had both the lowest sensitivity (39%) and the lowest accuracy (48%) among the evaluated classification schemes/parameters. Albumin gradient had the highest sensitivity (98%) but the lowest specificity (28%) to identify an exudate, while LDHef had the highest specificity (83%). The LDHef also had the highest accuracy (82%), followed by simplified Light’s criteria (79%). All 8/18 transudates misclassified as “exudates” via Light’s criteria (Table 2) occurred secondary to CHF and were classified as discordant exudates (based on TPr in 5/8 cats). Five of these eight cats received diuretics and one received an unknown injection prior to presentation and thoraco- (*n* = 5) or abdominocenthesis (*n* = 3). All eight transudates misclassified using Light’s criteria were also misclassified based on ALBg. In contrast, 6/47 exudates were misclassified as “transudates” using Light’s criteria (Table 2). Five of the six misclassified exudates were secondary to neoplastic disease, one was caused by cholangiohepatitis/triaditis. Of the six cats with exudates misclassified as “transudates”, four received corticosteroids, one received corticosteroids and non-steroidal anti-inflammatory drugs (NSAIDs), and one received NSAIDs prior to thoraco- (*n* = 2) or abdominocenthesis (*n* = 4).

### 3.3. Acute Phase Proteins in Effusions and Serum

**Figure 3:**
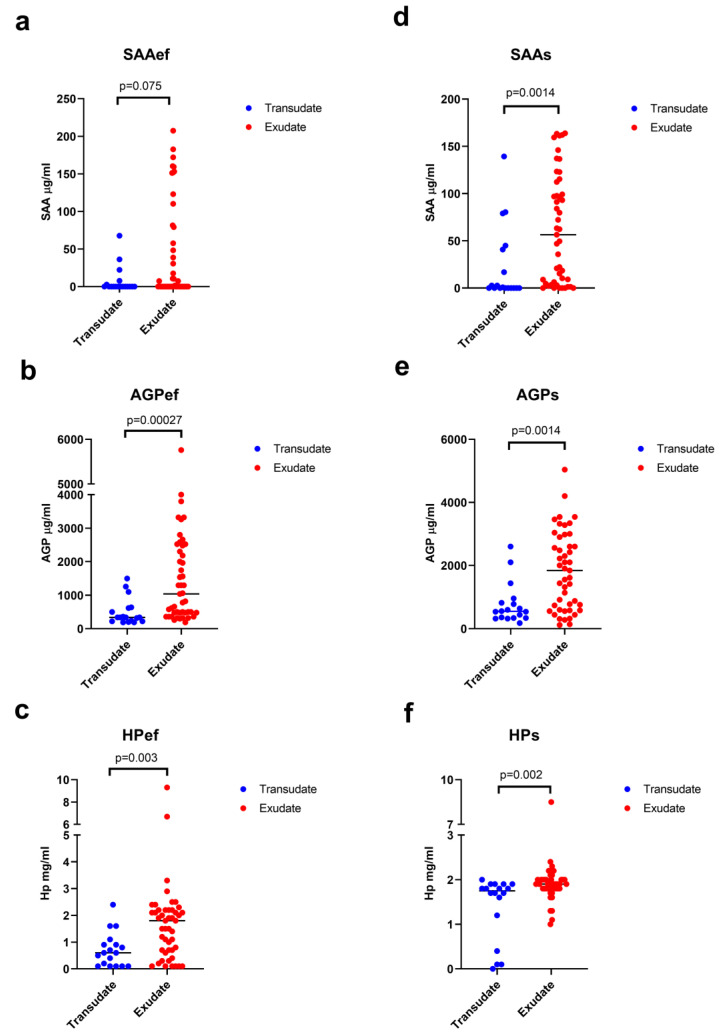
Dot plots showing comparison of concentrations of the acute phase proteins serum amyloid A (SAA), α1-acid glycoprotein (AGP), and haptoglobin (Hp) measured in effusion (SAAef, AGPef, HPef) (**a**–**c**) and serum (SAAs, AGPs, HPs) (**d**–**f**) of cats with transudates and exudates classified according to aetiological classification. Each cat/effusion is represented by a single blue (transudate) or red (exudate) dot. Horizontal lines indicate medians. *p*-values are given above the brackets.

Concentrations of APPs in both effusion and serum of cats with body cavity effusions were compared using aetiological classification and the traditional veterinary scheme (Table 3). There was a significant difference between cats with exudates and transudates for all APPs measured in effusion and serum apart from SAAef when aetiological classification was used (Table 3, Figure 3). With the exception of SAAef, the effect size was medium to large with Cohen’s d near or above 0.8 and the statistical power was near or above 80% (Table 3). However, APP concentrations did not differ between exudates and transudates when effusions were classified according to the traditional veterinary scheme. When the traditional veterinary scheme was used, sample effect size was small with a Cohen’s d of 0.1 to 0.45 and a low statistical power of 6% to 27% (Table 3).

**Figure 4:**
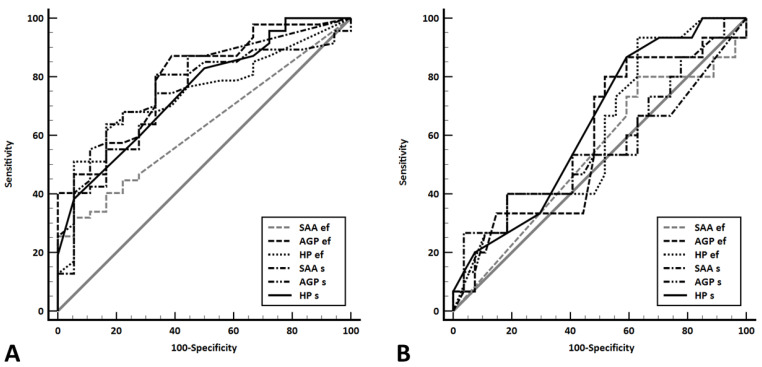
Receiver operating characteristic curves depicting the ability of the acute phase proteins (APPs) serum amyloid A (SAA), α_1_-acid glycoprotein (AGP), and haptoglobin (Hp) measured in both effusion (SAAef, AGPef, HPef) and serum (SAAs, AGPs, HPs) of 65 cats with body cavity effusions to differentiate between exudates and transudates classified according to aetiological classification (**A**) and the traditional veterinary scheme (**B**). Using aetiological classification, all APPs but SAAef could discriminate between exudates and transudates (**A**), but none of the APPs was discriminatory when effusions were classified according to the traditional veterinary scheme (**B**).

This lack of difference in APPs concentration when effusions were classified based on traditional veterinary scheme was reflected by the results of ROC analysis, with none of the APPs being able to differentiate between exudates and transudates (*p*-values for all AUCs > 0.05, Table 4, Figure 4). On the other hand, all APPs but SAAef could discriminate between the two effusion types when classified based on aetiology (Table 4, Figure 4).

Alpha-1-acid glycoprotein measured in effusion was the best parameter to differentiate between exudates and transudates based on aetiological classification, followed by AGPs and SAAs (Table 4, Figure 4). The best cut-off value for AGPef of >340 μg/mL had a sensitivity of 87% and a specificity of 61% in identifying an exudate. Using this cut-off value, AGPef correctly identified 11/18 (61%) transudates and 41/47 (87%) exudates. All seven transudates misclassified as exudates via AGPef were secondary to CHF. Four of those seven were correctly classified when using AGPs (at the cut-off value given in Table 4), the second best APP to differentiate between exudates and transudates. In the remaining three cats with misclassified transudates, AGPs (as well as AGPef) might have been increased due to causes other than CHF (fever of unknown cause documented during several days of hospitalisation [*n* = 1]; surgery for suspected intestinal foreign body a few days prior to presentation with CHF [*n* = 1]; endocarditis was confirmed via post-mortem examination as the cause of CHF [*n* = 1]). Exudates misclassified as transudates by AGPef were due to neoplasia (*n* = 3), pyothorax (*n* = 2) and idiopathic chylothorax (*n* = 1). The two cases with pyothorax were correctly classified as exudates via AGPs, but the remaining four effusions were not. Three of those four cats with exudates misclassified using AGPs (as well as AGPef) were treated with prednisolone prior to presentation.

## 4. Discussion

This study confirmed the superior diagnostic accuracy of Light’s criteria, simplified to include LDHef and TPr only, its components LDHef and TPr alone, and ALBg in differentiating exudates from transudates in comparison to the traditional veterinary scheme in a larger cohort of cats. Moreover, this study has also shown applicability of Light’s criteria, LDHef, TPr and ALBg to both pleural and peritoneal effusions, extending the use of these parameters to ascites. The ability of APPs in effusion and serum (apart from SAAef) to differentiate between exudates and transudates classified according to aetiological classification but not based on the traditional veterinary scheme further supports the superiority of aetiological classification over the traditional veterinary scheme.

Traditionally, body cavity effusions in veterinary medicine have been classified as transudates, modified transudates, and exudates based on TNCC and TP of the effusion [1]. However, this classification does not reflect the aetiology and pathogenesis of the effusion. A classification scheme based on aetiology has recently been reviewed by Dempsey and Ewing [39], and divided effusions into transudates, exudates, effusions resulting from vessel or viscus disruption, and effusions resulting from cell exfoliation. However, no clear cut-offs for biochemical parameters or guidance for diagnostic work-up was provided, and the classification likely could only be applied after making the diagnosis. One study adopted this approach, but it was unclear how, besides cytology, the diagnoses were made [40]. In human medicine, Light’s criteria have been used to classify pleural effusions as transudates or exudates, as well as guide diagnostic work-up, for over 50 years [8,41]. These criteria were also used [11] or adapted for use [42] with peritoneal effusions, although fewer reports exist. In cats, Light’s criteria have only been applied to pleural effusions to date [6,7].

In both of the previous feline studies, superior diagnostic accuracy of Light’s criteria in comparison to the traditional veterinary scheme has been shown [6,7]. In cats with pleural effusions, the traditional veterinary scheme had an accuracy of 40% [6] and 53% [7]. In the present study, including both pleural and peritoneal effusions, the accuracy levels lay between (48%) those two reported previously. It also should be noted that a number of effusions in previous reports (6/20 and 3/19) [6,7] as well as in the current investigation (23/65) could not be classified at all because their TNCC and TP did not fit any of the three categories. Light’s criteria had a diagnostic accuracy of 90% in the derivation cohort [6] and 84% in the validation cohort [7]. In the present report, the diagnostic accuracy of simplified Light’s criteria (omitting LDHr) in pleural and peritoneal effusions of 79% was only slightly lower than in the validation sample reported previously [7]. This result confirms the validity of the findings of previous studies in a larger cohort of cats, and supports the applicability of Light’s criteria to ascites as well. Additionally, given that simplified Light’s criteria omitting LDHr seem to have performed similarly to the full set of parameters used in previous research [6,7], the use of two criteria instead of the original three seems valid, and is cheaper. In human medicine, a meta-analysis of eight studies using Light’s criteria found that LDHef and LDHr were highly correlated and leaving out one of these from the triplet did not alter diagnostic accuracy [9].

As with any diagnostic test, the performance of the simplified Light’s criteria in the present report is dependent upon the cut-offs. While the cut-off for TPr used in this study was adopted from previous investigations [6,7], the laboratory’s own cut-off was used for the LDHef. The previously established LDHef cut-off [6,7] could not be used because the authors’ laboratory runs a different LDH assay. The laboratory’s own cut-off for LDHef of >194 IU/L was established with the aim of achieving the best sensitivity and specificity. If a cut-off based on the highest positive likelihood ratio would have been chosen (LDHef > 148 IU/L, see Supplementary Material S2), one additional cat would have had been correctly classified as having an exudate, increasing the accuracy of simplified Light’s criteria from 79% to 80%.

In both previous feline studies [6,7], only transudates were misclassified using Light’s criteria, which is similar to the situation reported in human medicine [10,41]. More transudates (8/18, 44%) than exudates (6/47, 13%) were misclassified in the current report; nevertheless, some exudates were also misclassified. All misclassified transudates were labelled as discordant exudates according to Light’s criteria and occurred secondary to CHF (only one transudate in this study was due to aetiology other than CHF). In human medicine, treatment with diuretics has been reported as the most common reason for misclassification of transudates using Light’s criteria and it was suggested that ALBg might be helpful in such cases [10,43]. In the present study, at least five of eight cats with misclassified transudates received diuretics (one additional cat received an unknown injection). However, all eight cases were also misclassified using ALBg. In a previous report, two of three cats with misclassified transudates secondary to CHF received diuretics, and in one of those two cats, ALBg correctly identified transudate [7]. Regarding the 6/47 exudates misclassified using Light’s criteria in the current study, 5 were due to neoplasia (lymphoma [*n* = 4], carcinoma [*n* = 1]), and 1 was caused by cholangiohepatitis/triaditis. It can be speculated that the composition of the effusion might have been affected by pre-treatment with anti-inflammatory drugs, leading to the misclassification of these exudates as transudates via Light’s criteria. Of these six cats, four received corticosteroids, one received corticosteroids and NSAIDs, and one received NSAIDs prior to thoraco- (*n* = 2) or abdominocenthesis (*n* = 4).

In addition to verifying the usefulness of Light’s criteria in a larger cohort of cats, one of the important aspects of the present investigation is the inclusion of a higher number of cats with FIP (14/65 [22%]; 3/31 [10%] pleural effusions) than in previous investigations. Only one cat with FIP was included in the previous derivation cohort [6], and there were none in the validation cohort [7]. In some recent studies, however, the prevalence of FIP was very similar to the present report (21/105 [20%] of pleural and peritoneal effusions [44]; 26/306 [8.5%] of pleural effusions [3]), making FIP an important differential for body cavity effusions in cats. Effusions caused by FIP are particularly interesting in terms of classification. Given their high protein content and relatively low TNCC, they are frequently classified as modified transudates, although, as stated by Pedersen [5], FIP effusions are “inflammatory exudates in the purest sense”. In the present study, 10 of 14 FIP effusions could not be classified at all using the traditional veterinary scheme and four were classified as modified transudates against their true exudative nature. However, Light’s criteria, LDHef, TPr and ALBg, all classified FIP effusions correctly, apart from one ascites misclassified via TPr as transudate. These findings further support the usefulness and validity of these classification parameters.

The second objective of this investigation was to assess the value of the measurement of serum and effusion APPs in differentiating between exudates and transudates using aetiological classification and the traditional veterinary scheme. In human medicine, C-reactive protein (CRP) measured in both serum and pleural effusion [45], SAA in pleural and peritoneal effusion [46], and AGP [47] and ceruloplasmin [48,49] in pleural effusion have all been shown to discriminate between exudates and transudates. In cats, APPs have been shown to increase with inflammation, trauma or neoplasia [16,17,18,50]; to date, however, only one study has assessed their concentrations in effusions. That study identified higher AGP concentrations in cats with FIP in comparison to healthy cats and cats with other diseases causing effusions, while HP was not discriminatory [15]. Alpha-1-acid glycoprotein measured in the effusion was also the best APP to differentiate between FIP and other diseases in our previous study, using cats that were also included in the current report [22]. Whether APPs measured in effusion or serum can help differentiate between exudates and transudates was not assessed in previous feline reports [15,21]. Given the findings of the above-mentioned research in humans and the behaviour of APPs in cats with FIP, which as an exudative condition, we expected to detect higher concentrations of APPs in effusion and serum in cats with exudates. Interestingly, the concentrations of APPs in both serum and effusion (apart from SAAef) differed significantly between cats with exudates and those with transudates when classification was made using the aetiological scheme but not according to the traditional veterinary approach. This conformity of APPs with aetiological classification but not the traditional one can be interpreted as another piece of evidence in favour of the aetiological classification.

Both serum and effusion APPs were evaluated in this study as in clinical practice it sometimes might be easier to obtain one sample over the other and we were therefore interested in identifying the most useful sample/APP. For example, when only a small amount of effusion is present, it might not be readily accessible, and serum might be preferred. On the other hand, in debilitated animals, the blood volume that can be drawn might not be sufficient to perform all diagnostic tests. In addition, financial resources might not always allow for multiple tests. In humans, some serum APPs have been shown to correlate better with their effusion counterparts than others, and differences in their ability to discriminate exudative from transudative effusions exists [46]. Therefore, three APPs were measured in both serum and effusions from cats, and their ability to differentiate between exudates and transudates was assessed via ROC analysis. Using the aetiological classification, SAAef was not discriminatory; however, there was no significant difference in the discriminatory ability of the other APPs (based on a comparison of the AUCs for these parameters). Numerically, AGPef had the largest AUC (0.79), followed by AGPs (0.76) and SAAs (0.76). Therefore, where availability of the test is not an issue, AGP in effusion and preferably also AGP in serum should be measured to aid in differentiating exudates from transudates. However, there are some issues with the availability of the AGP assay (currently, only an AGP ELISA is commercially available), while the measurement of SAA is widely available in commercial laboratories and in in-house circumstances. Therefore, the assessment of SAA in serum might be the best option for many clinicians. The cut-offs for AGPef, AGPs or SAAs used in this study, however, might not be transferable when other assays are used to measure these APPs than those employed in the present study. Although the feline AGP ELISA was recently validated in our laboratory, due to the unavailability of the SRID assay, correlation and bias between the ELISA and SRID assay could not be determined [51]. Similarly, a new cut-off will be needed when using the new improved format of the LZ Test ‘Eiken’ SAA (marketed as VET-SAA). This test uses purely monoclonal antibodies, and is supposed to be more specific and less prone to variation between assay batches [52].

Although AGPef was the best parameter to differentiate between exudates and transudates in this study, the concurrent measurement of AGP in serum or SAA in serum might be helpful. In fact, four of the seven misclassified transudates based on AGPef were correctly classified using AGPs. In the remaining three cats, the high AGPs and AGPef could be explained by conditions other than CHF, which was clearly the cause of the effusion in those cases. One of those three cats had fever documented during several days of hospitalisation (cause unknown), another cat had a surgery for suspected intestinal foreign body a few days prior to presentation with CHF, and the third cat had endocarditis (confirmed via post-mortem examination) as the cause of CHF. Among the six exudates misclassified based on AGPef, two (both pyothorax) were correctly classified using AGPs. In the remaining four cats, three were treated with prednisolone prior to presentation, which might have influenced APP concentration. These misclassified cases demonstrate the limitations of using APPs to differentiate between exudates and transudates. Nevertheless, the measurement of APPs, especially AGP, has proven useful in most cases. In future studies, as well as in clinical practice, the possible effect of comorbidities (e.g., surgery or an inflammatory condition in a cat with CHF causing effusion) and prior anti-inflammatory treatment needs to be considered when interpreting APPs concentrations.

There are some limitations to this study. First, the Light’s criteria could not be assessed in full, because LDH was only measured in effusion but not in serum. Therefore, LDHr, which is a part of Light’s criteria, could not be assessed in this investigation. However, some authors criticised the concurrent use of LDHef and LDHr as they are not independent of each other [9]. Therefore, the sole use of LDHef might only represent a minor limitation, especially given the results of diagnostic accuracy of the simplified Light’s criteria, which was similar to a previous investigation [7]. Furthermore, given the lack of RR for serum LDH in the authors’ laboratory, it was not possible to calculate the LDHef cut-off as two-thirds of the normal upper limit for serum LDH. The latter approach was used in some studies in humans [32] to account for differences in LDH assays across laboratories [29]. Studies in cats are needed to determine whether this cut-off is applicable to feline effusions as well. Using the LDHef cut-off as two-thirds of the normal upper limit for serum LDH might facilitate a wider use of LDHef in routine laboratory analysis of effusion and aid effusion classification.

Another limitation is that the number of cats included in this study was limited by the availability of suitable cases within the timeframe of the study and was not based on a power calculation. The retrospective power calculation revealed medium-to-large sample effect sizes and sufficient power of the aetiological classification to differentiate between exudates and transudates via APPs in blood and effusion. Nevertheless, a small sample effect size and low statistical power were detected for the traditional veterinary scheme.

A further limitation is that post-mortem examination confirming the final clinical diagnosis, and consequently the aetiological classification of the effusion used as “gold standard”, was only performed in a proportion of cases included in this study (*n* = 20). However, for cardiac disease or neoplasia, which represented most diseases in this report, clinical diagnosis is usually straightforward. Diagnosis of FIP might be challenging; however, the machine learning algorithm used to identify cats with FIP was trained on cats with immunohistochemistry-confirmed FIP and all cats included in the study had multiple tests performed with respect to FIP, the results of which were used to “feed” the algorithm [21]. Another limitation is that only one pericardial effusion was included and therefore conclusions about applicability of the simplified Light’s criteria, ALBg or APPs to pericardial effusions cannot be drawn from this investigation. Additionally, all but one transudate was secondary to CHF; therefore, the applicability to transudates arising from reduced oncotic pressure could not be assessed. However, such transudates are rare in cats and were not described in previous studies either [3,6,7,43]. It is therefore not surprising that no such cats were presented within the timeframe of this investigation. Finally, some cats were treated with anti-inflammatory drugs or diuretics prior to presentation, which might have affected the diagnostic test results. This, unfortunately, is a common limitation of studies at referral centres because cats mostly present after being initially assessed or even treated at their primary veterinarians. Future studies should be designed to avoid the inclusion of cats that have already received such treatments.

## 5. Conclusions

In conclusion, this study could confirm the superior diagnostic accuracy of simplified Light’s criteria, as well as that of its components LDHef and TPr, and ALBg in differentiating exudates and transudates in cats in comparison to the traditional veterinary scheme. The applicability of these parameters to ascites, in addition to pleural effusion, could be demonstrated. The study also revealed that APPs correspond better with the aetiological classification of effusions in comparison to the traditional veterinary scheme. Alpha-1-acid glycoprotein measured in effusion was found to be the best parameter to differentiate between exudates and transudates. Although neither effusion nor serum APPs can replace the routine laboratory and cytological assessment of the body cavity effusions, the measurement of APPs in serum and effusion, especially AGP, represents a useful additional tool in aiding classification and guiding diagnostic tests.

## Figures and Tables

**Table 1:** Classification of effusions in 65 cats based on localisation (pleural, pericardial, peritoneal) and aetiology.

Transudate (*n* = 18)			Exudate (*n* = 47)	
Pleural effusion (*n* = 13)	Pericardial effusion (*n* = 1)	Ascites (*n* = 4)	Pleural effusion (*n* = 18)	Ascites (*n* = 29)
CHF (*n* = 13)	CHF (*n* = 1)	CHF (*n* = 3) AKI, ureteric obstruction (*n* = 1)	Tumour (carcinoma [*n* = 4], lymphoma [*n* = 4], other [*n* = 1]) Pyothorax (*n* = 4) FIP (*n* = 3) Idiopathic chylothorax (*n* = 2)	FIP (*n* = 11) Tumour (lymphoma [*n* = 6], carcinoma [*n* = 5], other [*n* = 1]) Septic peritonitis (*n* = 4) Cholangiohepatitis/triaditis (*n* = 1) Haemoabdomen due to trauma (*n* = 1)

CHF—congestive heart failure; AKI—acute kidney injury; FIP—feline infectious peritonitis.

**Table 2:** Sensitivity, specificity, and accuracy to identify an exudate, and number (proportion) of misclassified transudates and exudates based on the traditional veterinary scheme, lactate dehydrogenase activity in effusion (LDHef), effusion/serum total protein ratio (TPr), simplified Light’s criteria (LDHef, TPr) and serum–effusion albumin gradient (ALBg) when compared to the gold standard aetiological classification of effusions (18 transudates, 47 exudates). A total of 65 (33 peritoneal, 31 pleural, 1 pericardial) effusions were included. The highest sensitivity, specificity, and accuracy is in bold.

Classification Scheme	Sensitivity	Specificity	Accuracy	Misclassified Transudates *n* (%)	Misclassified Exudates *n* (%)
Traditional veterinary scheme *^#^	39%	73%	48%	3/11 (27%)	19/31 (61%)
LDHef (IU/L)	81%	**83%**	**82%**	3/18 (17%)	9/47 (19%)
TPr	79%	72%	77%	5/18 (28%)	10/47 (21%)
Simplified Light’s criteria	87%	56%	79%	8/18 (44%)	6/47 (13%)
ALBg	**98%**	28%	75%	13/18 (72%)	1/47 (2%)

* Modified transudates are considered transudates for the purpose of this analysis. ^#^ 42 effusions that could be classified are included in the calculation.

**Table 3:** Comparison of concentrations of the acute phase proteins (APPs) serum amyloid A (SAA), α_1_-acid glycoprotein (AGP), and haptoglobin (Hp) measured in effusion (SAAef, AGPef, HPef) and serum (SAAs, AGPs, HPs) of cats with transudates and exudates based on aetiological classification of the effusion and traditional veterinary scheme. Effusion and serum samples of 65 cats with peritoneal (*n* = 33), pleural (*n* = 31), and pericardial (*n* = 1) effusions were used. Data are presented as median (range), with sample effect size (Cohen’s d) and power (1-β error probability). Significant *p*-values, large sample effect size (d ≥ 0.8), and sufficient power (≥80%) are in bold.

Classification Scheme	APPs	Transudate	Exudate	*p*-Value	Cohen’s d	Power%
Aetiological classification		*n* = 18	*n* = 47			
	SAAef [μg/mL]	0.1 (0.1–67.7)	0.1 (0.1–207.4)	0.075	0.40	28
	AGPef [μg/mL]	340 (190–1500)	1040 (190–5760)	**0.00027**	0.79	78
	HPef [mg/mL]	0.6 (0.1–2.4)	1.8 (0.1–9.3)	**0.003**	0.79	78
	SAAs [μg/mL]	0.45 (0.1–139.2)	56.4 (0.1–163.8)	**0.0014**	**0.86**	**85**
	AGPs [μg/mL]	550 (180–2600)	1840 (120–5040)	**0.0014**	**0.86**	**85**
	HPs [mg/mL]	1.75 (0–2)	1.9 (1–8.5)	**0.002**	**0.82**	**82**
Traditional veterinary scheme *		*n* = 27	*n* = 15			
	SAAef [μg/mL]	0.1 (0.1–159.4)	0.1 (0.1–182.7)	0.71	0.10	6
	AGPef [μg/mL]	580 (190–3260)	480 (190–3800)	0.36	0.28	13
	HPef [mg/mL]	0.8 (0.1–2.5)	1 (0.1–2.2)	0.25	0.37	19
	SAAs [μg/mL]	15.3 (0.1–162.1)	35.8 (0.1–163.8)	0.65	0.13	7
	AGPs [μg/mL]	820 (140–3540)	780 (120–3320)	0.56	0.18	8
	HPs [mg/mL]	1.8 (0–2.2)	1.9 (1.3–2.3)	0.15	0.45	27

* Modified transudates are considered as transudates for the purpose of this analysis.

**Table 4:** Areas under the curve (AUCs), their confidence intervals (CI), optimal cut-off values and their sensitivities and specificities to differentiate between exudates and transudates according to aetiological classification and the traditional veterinary scheme for the acute phase proteins (APPs) serum amyloid A (SAA), α1-acid glycoprotein (AGP), and haptoglobin (Hp) measured in both effusion (SAAef, AGPef, HPef) and serum (SAAs, AGPs, HPs) of 65 cats with body cavity effusions (33 peritoneal, 31 pleural, 1 pericardial). Significant *p*-values for AUCs are in bold. AUCs that are significantly different from each other within the two classification schemes are marked with # or §.

Classification	APPs	AUC (95% CI)	*p*-Value	Best Cut-Off (Sensitivity%, Specificity%)
Aetiological classification	SAAef ^#§^ [μg/mL]	0.63 (0.5–0.75)	0.07	>36.2 (Sens. 32%, Spec. 94%)
	AGPef ^#^ [μg/mL]	0.79 (0.68–0.88)	**<0.0001**	>340 (Sens. 87%, Spec. 61%)
	HPef [mg/mL]	0.74 (0.61–0.84)	**0.0002**	>0.9 (Sens. 68%, Spec. 78%)
	SAAs [μg/mL]	0.76 (0.63–0.85)	**0.0001**	>2.7 (Sens. 81%, Spec. 67%)
	AGPs ^§^ [μg/mL]	0.76 (0.64–0.85)	**<0.0001**	>960 (Sens. 64%, Spec. 83%)
	HPs [mg/mL]	0.75 (0.63–0.85)	**0.0001**	>1.7 (Sens. 83%, Spec. 50%)
Traditional veterinary scheme *	SAAef [μg/mL]	0.53 (0.37–0.69)	0.75	≤7.5 (Sens. 80%, Spec. 37%)
	AGPef [μg/mL]	0.59 (0.42–0.74)	0.36	≤620 (Sens. 80%, Spec. 48%)
	HPef [mg/mL]	0.61 (0.45–0.76)	0.23	≤1.6 (Sens. 93%, Spec. 37%)
	SAAs [μg/mL]	0.54 (0.38–0.7)	0.68	>122.9 (Sens. 27%, Spec. 96%)
	AGPs [μg/mL]	0.55 (0.39–0.7)	0.57	≤440 (Sens. 40%, Spec. 82%)
	HPs [mg/mL]	0.64 (0.47–0.78)	0.12	>1.7 (Sens. 87%, Spec. 41%)

* Modified transudates are considered as transudates for the purpose of this analysis. ^#^ AUCs are significantly different, with *p* = 0.0043; ^§^ AUCs are significantly different, with *p* = 0.045.

## Data Availability

Data are contained within the article or Supplementary Material.

## Supplementary Materials

The following supporting information can be downloaded at: https://www.mdpi.com/article/10.3390/ani13121918/s1, Supplementary Material S1: diagnostic test results of all 65 cats; Supplementary Material S2: effusion lactate dehydrogenase cut-off establishment.
